# Revealing the interference effect of Majorana fermions in a topological Josephson junction

**DOI:** 10.3762/bjnano.9.50

**Published:** 2018-02-12

**Authors:** Jie Liu, Tiantian Yu, Juntao Song

**Affiliations:** 1 Department of Applied Physics, School of Science, Xi’an Jiaotong University, Xi’an 710049, China; 2 Department of Physics and Hebei Advanced Thin Film Laboratory, Hebei Normal University, Shijiazhuang 050024, China

**Keywords:** density of states, fractional Josephson effect, Majorana fermion

## Abstract

We study theoretically the local density of states (DOS) in a topological Josephson junction. We show that the well-known 4π Josephson effect originates from the interference effect between two Majorana fermions (MFs) that are localized at the Josephson junction. In addition, the DOS for electrons (holes) shows the 4π interference information along each parity conserved energy spectrum. The DOS displays a 2π period oscillation when two trivial states interfere with each other. This means that the DOS information may be used to distinguish the MFs from trivial localized states. We suggest that the interference effect and the DOS can be detected by using two STM leads or two normal leads. A single side lead can only detect the Andreev reflection tunneling process in the junction, which cannot reveal information about the interference effect in general. However, using two side leads, we can reveal information about the interference effect of the MFs as well as the DOS by combining Andreev reflection with the electron transmission process.

## Introduction

After Kitaev reported that Majorana fermions (MFs) can appear as quasi-particle states at the ends of a one-dimensional (1D) p-wave superconductor [1], the generation of MFs became a popular goal in condensed matter physics [2]. Several methods were suggested to fabricate and detect MFs in effective 1D p-wave superconductor systems [3–11]. The use of a semiconductor wire with Rashba spin–orbit coupling and proximity-induced superconductivity appear to be the most promising method [4]. Indeed, a semiconductor–superconductor nanowire was manufactured to confirm the prediction of the theory [12–14]. The second topological superconducting system that was realized experimentally is related to ferromagnetic atomic chains, which are put on a trivial superconductor [15]. It is believed that MFs can generate a zero-bias conductance peak (ZBP) in the conductance spectrum [16–19], and indeed the signature of ZBPs has been observed in both systems in tunneling experiments. These advances accelerate the development of nanotechnology [20–27]. Recently, a breakthrough was achieved in research groups led by Kouwenhoven and Marcus. Both groups observed the integer ZBPs in a nanowire system [28]. These are the most persuading results so far. However, all these achievements relied on the observation of ZBPs, which means that many other unique properties of MFs still require further verification and investigation.

Apart from the ZBP, another significant feature of MFs is the 4π Josephson current. When two topological superconducting wires are combined to form a topological Josephson junction (Top-JJ), the period of the supercurrent is 4π if MFs exist at the ends of both wires. This is different from the trivial case without MFs. In the trivial case in which only Cooper pairs can tunnel, the period is 2π. Since MFs have only half a degree of usual fermions, half a degree of Cooper pairs can tunnel in the Top-JJ when the two MFs combine. In this situation, the period is doubled. Because the 4π Josephson effect is a unique transport property of MFs, many groups attempt to observe it. Indeed, Kouwenhoven’s and Marcus’ groups fabricated such a junction and obtained some preliminary results. However, the expected 4π period was not observed [23–25]. The 4π Josephson effect needs a stringent condition that is known as the parity conservation [29]. The evolution of the states is expected to follow one fixed branch of the energy spectrum. It is particularly susceptible at the degenerate point when the even and the odd parity states intersect at zero energy for 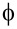 = (2*n* + 1)π. The state then changes from one parity to another because of quasiparticle poisoning, the background and the thermal effect [30–34]. In this case, the 4π period will return to the conventional 2π. Thus, to reveal the 4π nature of the MFs, it may be necessary to observe more than just a supercurrent. Interestingly, several groups have studied superconductor-topological insulator–superconductor junctions that also display a 4π Josephson current. However, the behavior of the 4π Josephson current is not consistent with the theoretical prediction [35–41]. To distinguish the 4π information of MFs, it is necessary to reveal additional characteristic properties of such a Josephson junction.

**Figure 1:**
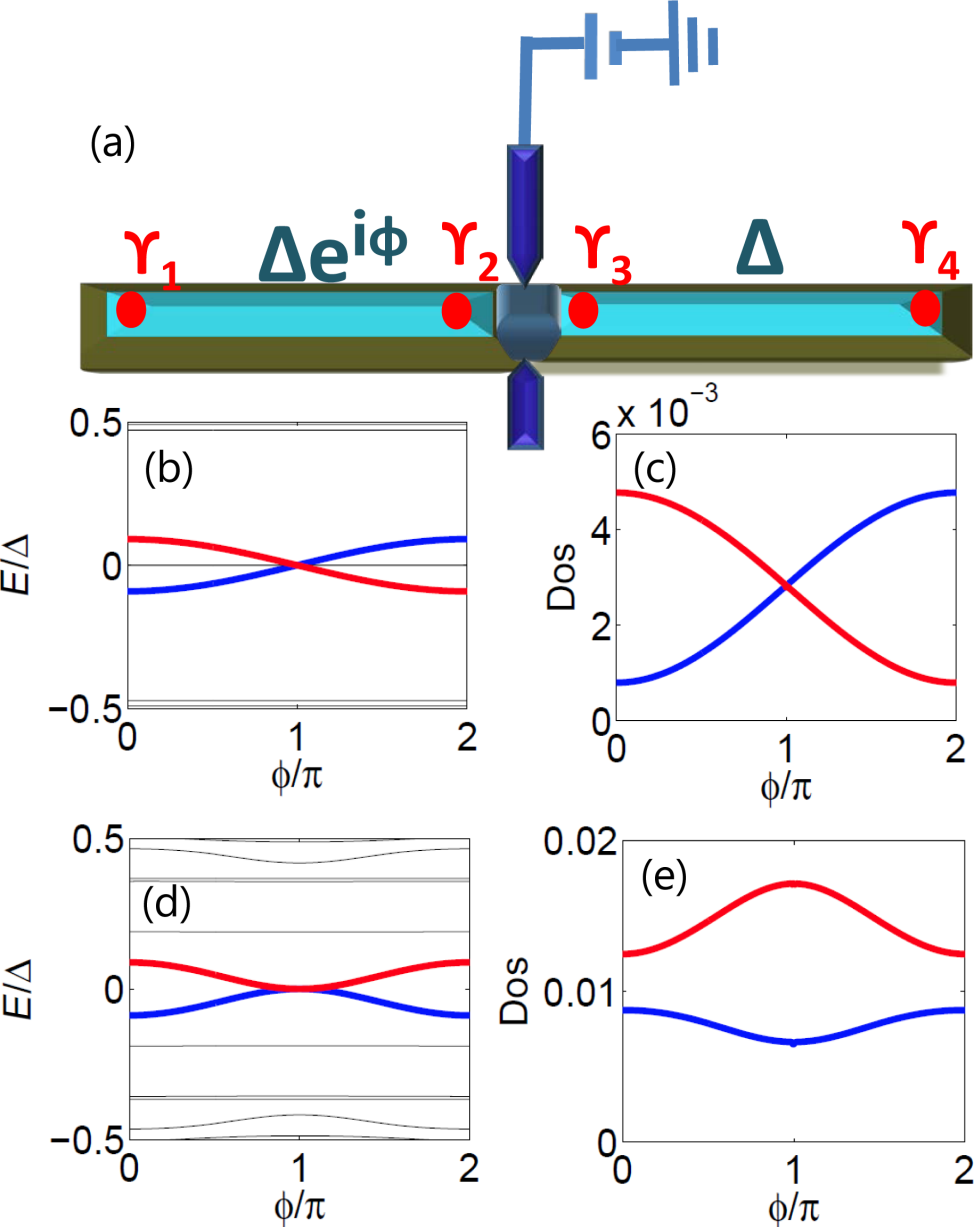
(a) Schematic setup of an experiment in which two STM leads or normal leads are connected to a Top-JJ that supports the MFs. (b) Energy spectrum of the Top-JJ with chemical potential μ = −2*t*, which lies in the topological region. The two MFs, which are localized at the junction, interfere with each other and display a 4π oscillation. (c) DOS for electron part of the coupled MFs in the Top-JJ. Both even parity state and odd parity state show a parity-correlated 4π oscillation. (d) Energy spectrum of the Top-JJ with chemical potential μ = −2*t* + 5.7Δ, which lies in the trivial region, and the disorder strength **w** = 0.13*t*. In this case, there does not exist any MF that is localized at the junction. However, the trivial Andreev bound states occasionally touch with each other in the presence of disorder. In such situation, the trivial Andreev bound states behave like the Andreev bound states formed by the two MFs in panel (b). (e) DOS of the trivial Andreev bound states for the electron part. It is totally different from the DOS of the nontrivial Andreev bound states in panel (c). The period of the trivial state is 2π.

In this paper, we study a Top-JJ composed of two topological superconductors as shown in Figure 1a. Unlike previous studies, we focus on the density of states (DOS) for both the electron part and the hole part. The essential property of the MFs is that the wave function of the electron part must be conjugated with the wave function of the hole part, which is known as the self-Hermitian property of the MFs. More specifically, the self-Hermitian property of the MFs can be demonstrated directly from the DOS of the electron and of the hole part, which is a basic assumption used in this paper. Since the DOS only shows the steady information of the whole energy spectrum, it does not relate to the parity-conserving problem, which is a problem of dynamic evolution. Therefore, compared to the supercurrent, the DOS are easier to detect. We show that the two Andreev bound states formed by the MFs exhibit a 4π period due to the interference effect between the two MFs. Furthermore, the DOS of both the electron and the hole part can also reveal the 4π period. The electron (hole) DOS of the two Andreev bound states are related: One is destructive, while the other is constructive. However, the DOS of the trivial Andreev bound states contains different information. In general, the interference effects in the trivial Andreev bound states are unrelated, and their period is 2π. Thus, it may be a way to distinguish them using information contained in the DOS. We suggest that the interference effect can be detected using two STM leads or two normal leads. We show that a single side lead can only detect the Andreev reflection tunneling process in the junction, which cannot reveal information about the interference effect in general. However, using the two side leads, we can display information about the interference effect of the MFs by combining Andreev reflection and the electron-transmission process.

## Model Hamiltonian and formula

A typical Top-JJ is composed of two topological superconducting wires that have different superconducting phases. According to [9,18], the tight-binding model of a superconducting wire is:

[1]
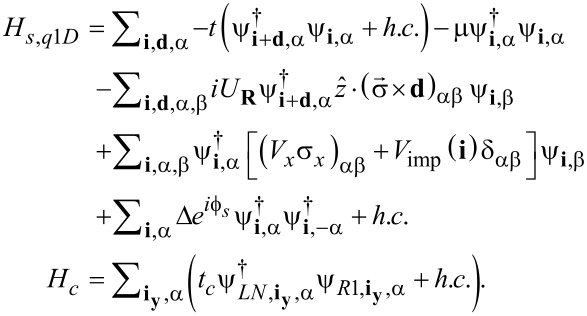


Here, *H_s,q1D_* is the Hamiltonian of the left (right) wire with *s* = *L* (*R*). The only difference between the two wires is the phase of the superconducting order 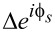 (here we set 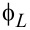 = 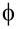 and 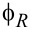 = 0). Furthermore, **i** denotes the lattice site, and **d** denotes the two unit vectors **d_x_** and **d_y_**, which connect the nearest neighbor sites in the *x* and *y* directions, respectively. Moreover, α, β are the spin indices, *t* is the hopping amplitude, μ is the chemical potential, *U_R_* is the Rashba coupling strength, and *V_x_* is the Zeeman energy caused by magnetic field along the wire direction. Δ is the superconducting pairing amplitude and *V*_imp_(**i**) is the Gaussian impurity. *H_c_* describes the coupling between the left and the right topological superconducting wires.

To obtain the tunneling coefficient at the junction, we use the recursive Green function method. We can then calculate the scattering matrix of the system. The scattering matrix is related to the Green functions via

[2]
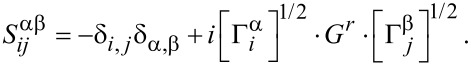


Here, 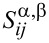 is an element of the scattering matrix that denotes the scattering amplitude of a β particle from the *j*-th lead to an α particle in the *i*-th lead. Furthermore, *i*,*j* = 1 or 2, where 1 and 2 denote, respectively, the first and the second normal lead as shown in Figure 1a. 
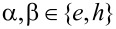
 denote the electron (*e*) or hole (*h*) channels. In addition,


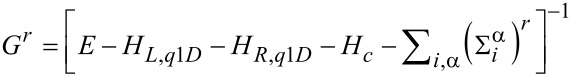


is the retarded Green function of the Josephson junction, and 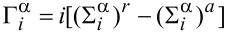 is the linewidth function of an α particle in the *i*-th lead, where 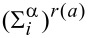 is the retarded (advanced) self-energy of the α particle for the *i*-th lead. In the following calculation we set 
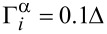
 through wide-band approximation. The physical meaning of the scattering matrix is: 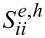 means the Andreev reflection coefficient *T_A_* in the *i*-th lead, and 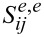 means the electron transmission coefficient *T_e_* from the *i*-th lead to the *j*-th lead.

To match the experiment in [12], the parameters in the tight-binding model were chosen as follows: Δ = 250 μeV, *t* = 25Δ, *U_R_* = 2Δ, and the superconductor coherence length is ξ = *t*/Δ*a* = 500 nm with *a* being the lattice constant. In addition, we set *V_x_* = 2Δ such that the superconducting wire can support the MF end states by tuning the chemical potential.

## Results and Discussion

The following section is divided into three subsections. In the first subsection, the 4π oscillation of the DOS is shown. In the second subsection, the same oscillation information in a ring structure is shown and in the third subsection, we discuss how the information about the DOS is detected.

### 4π oscillation of the density of states

In this subsection, we consider the origin of the 4π Josephson effect. Then, we show that the DOS for the electron (hole) part can also exhibit the 4π interference effect. The well-known 4π Josephson effect is directly related to the fractional nature of the MFs. Because a single MF has only half a degree of a conventional fermion, we can define a conventional fermion using ψ*_j_* = (γ*_2j−1_* + *i*γ*_2j_*). For the Top-JJ in Figure 1a, there are two pairs of the MFs, which are localized at the ends of the superconductor. We assume that the length of the wire is sufficient so that γ_1_ and γ_2_ (γ_3_ and γ_4_) are not coupled to each other. In this case, only γ_2_ and γ_3_ can couple to each other at the junction, which is described in Equation 1. Because the phase of the left wire is 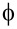 and the phase of the right wire is 0, the Hamiltonian of the left wire can be transformed into the right one using a unitary transformation 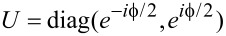. The phase difference between γ_2_ and γ_3_ is 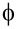/2. These two MFs will interfere with each other and form two Andreev bound states because of this phase difference. The effective Hamiltonian can be obtained by projecting the coupling of Equation 1 onto the subspace of the MFs using 
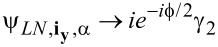
 and 
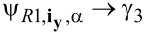
[6]. Then, the low-energy effective Hamiltonian is

[3]
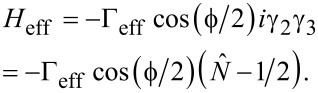


Here, 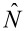 = ψ^†^ψ is the number operator and ψ = γ_3_ + *i*γ_2_. Then, the occupation number has two values: *N_v_* = 0,1, with *N_v_* = 0 corresponding to the even parity state, and *N_v_* = 1 corresponding to the odd parity state. The Josephson current mediated by the MFs can be given by 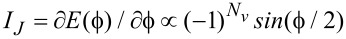, which displays the 4π oscillation. This is very different from the case without the MFs. In such case, only Cooper pairs can tunnel from one superconductor to another, and the period is 2π.

We show that the fractional Josephson effect can be attributed to the interference effect between the two MFs. Next, we show that the DOS of the electron and the hole part of the Andreev bound states, which are formed by the MFs, also display the 4π period. The MF is a particle that is its own antiparticle. For such a particle, the wave function of the electron part must be conjugated with the wave function of the hole part, which is the self-Hermitian property of the MF. Thus, the general wave function of the MFs should be [42]:


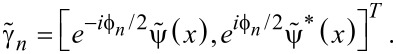


Here, 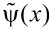 is the wave function of the electron part, when the phase of the superconducting order parameter is 0. In the Top-JJ shown in Figure 1(a),


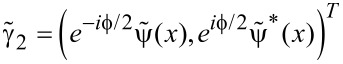


and


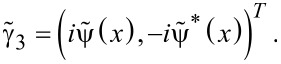


These two degenerate MFs will couple with each other to form an Andreev bound state via ψ = (γ_3_ + *i*γ_2_), and the excited wave function should be combined using the same rule:

[4]
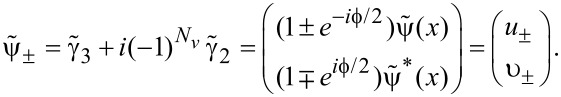


From Equation 4 we can see that the DOS for the electron part is 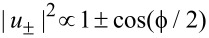, while the DOS for the hole is 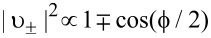. There are several unique properties of the DOS for the Andreev bound states formed by the MFs: First, the period along each energy spectrum is 4π. Second, it is parity correlated. The DOS is 
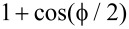
 for the even parity state, and the DOS is 
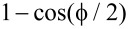
 for the odd parity state. Third, the DOS of the hole part for the even parity state is the same as that of the electron part for the odd parity state due to the self-Hermitian property of the MFs. Because of these unique properties of the DOS, we may differentiate the 4π information using the DOS of the electron (hole) part, which should provide clearer distinctions than the trivial states.

Our numerical results provide direct evidence for this conclusion. We use the tight-binding model in Equation 1. The length of each wire is *N_x_a* = 4μm and *t_c_* = 0.4*t*. Figure 1b shows the energy spectrum as a function of the flux 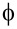 with the chemical potential μ = −2*t*, which lies in the topological region. The red solid line is the energy spectrum for the odd parity state, while the blue solid line is the energy spectrum for the even parity state. We can see that both of them oscillate with a period of 4π. Next, we study the information of the DOS more closely. Figure 1(c) shows the information of the local DOS for the electron part 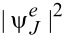 along the fixed even parity state (blue solid line) and the odd parity state (red solid line). Here, 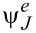 is the electron part wave-function localized at the junction, which can be extracted through diagonalization of the lattice Hamiltonian in Equation 1. The DOS of the electron oscillates with a period of 4π and the interference pattern is correlated with the parity. Furthermore, this relation is still valid in the presence of moderate disorder. Figure 1b and Figure 1c are calculated for the Gaussian disorder of *w* = 0.06*t*. We can see that the relation still holds.

Interestingly, when the two trivial fermion states interfere with each other, the situation is very different. Though an analytic result cannot be obtained, our numerical simulation suggest that the general formula for the DOS for the electron (hole) part should be 
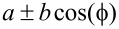
, with *a* and *b* being real constants. This can be understood as follows: For the trivial case, only Cooper pairs can tunnel through the junction. Thus, the DOS must be a function of cos(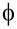) instead of cos(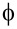/2). From our numerical results, we know there are several differences to the nontrivial case. First, the period is 2π. Second, there is no corresponding parity-correlated interference effect for the trivial case. Third, the maximum (minimum) value of the DOS is at 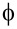 = (2*n* + 1)π for the trivial case and at 2*n*π for the nontrivial case. In Figure 1d, we show the energy spectrum as a function of the flux under strong disorder, *w* = 0.13*t* with μ = −2*t* + 5.7Δ. It is typical that the two trivial Andreev bound states are accidentally in contact with each other for the strong disorder. From Figure 1b and Figure 1d, we can see that the energy spectra are very similar between the trivial case without the MFs and the nontrivial case with the MFs. In this situation, it is difficult to distinguish the trivial Andreev bound states from the Andreev bound states formed by the MFs. Even though the period of the Josephson current is still 2π, it may be changed into 4π via a Landau–Zener transition [43]. Thus, the Josephson current cannot distinguish the trivial Andreev bound states and the nontrivial Andreev bound states formed by the MFs. Figure 1e displays the information of the DOS for the electron part for the two trivial Andreev bound states. We can see that the DOS is described by 
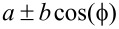
, which is distinct from the nontrivial case shown in Figure 1(c). Therefore, the DOS are clearly distinct.

### Interference effect in a ring structure

**Figure 2:**
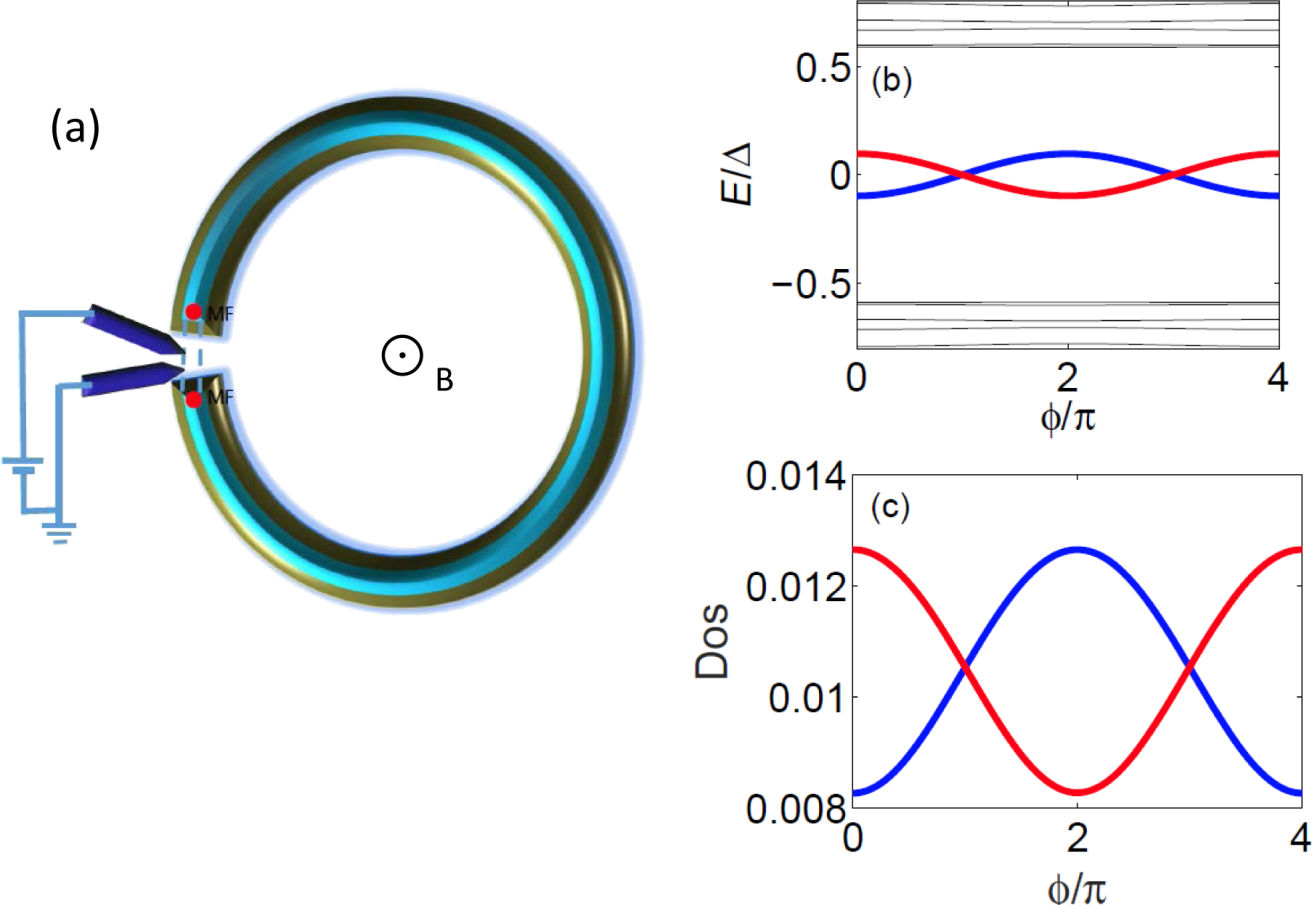
Interference effect in a typical Top-JJ of a ring structure. (a) Schematic setup of the experiment. (b) Energy spectrum of the Top-JJ with the chemical potential μ = −2*t* which lies at the topological region. The two MFs which are localized at the junction interfere with each other and display the 4π oscillation. (c) DOS for the electron part of the coupled MFs in the Top-JJ. Both the even parity and the odd parity states show parity correlated 4π oscillation.

Another typical Josephson junction is the ring structure shown in Figure 2a. In such a ring structure, when a magnetic flux threads the ring, the two MFs interfere with each other due to the phase difference. In Figure 2b, we show the energy spectrum as a function of the flux. The Andreev bound states formed by the two MFs show the same behavior as for the Top-JJ shown in Figure 2b. Furthermore, the DOS of the electron part in Figure 2c also contains the same interference information as the one shown in Figure 2c. They are parity correlated with a 4π period. Thus, we can see that the fractional Josephson effect originates from the interference effect between the two MFs.

**Figure 3:**
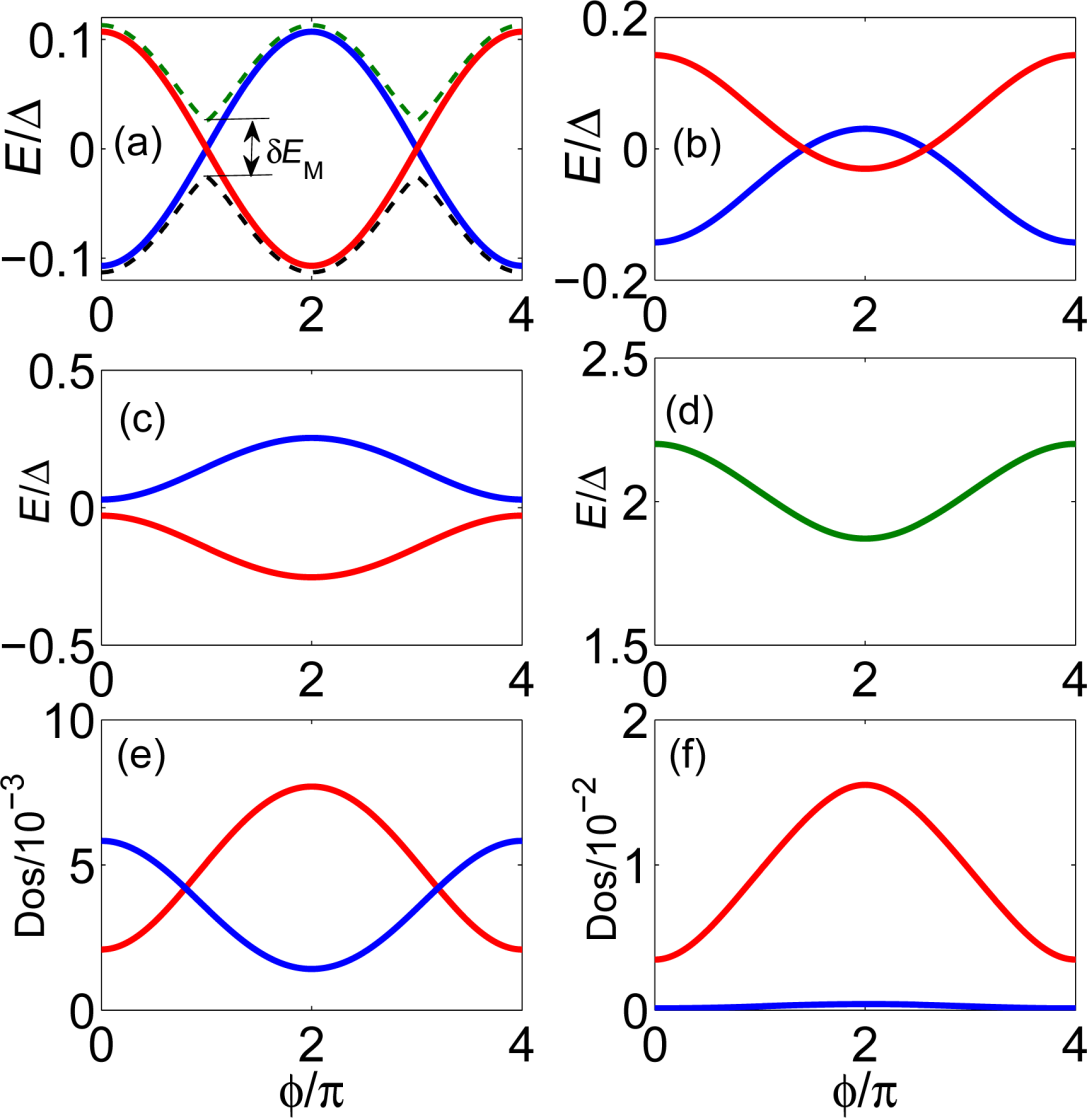
(a) For the Top-JJ shown in Figure 1a, when we consider the energy splitting induced by the finite length of the wire, the parity will be destroyed. The dashed line shows the energy spectrum versus the phase difference with *L*_1_ = *L*_2_ =100*a*. A small gap δ*E_M_* can be observed due to the finite-length effect. (b) Energy spectrum as a function of the flux in the ring structure with μ = −2*t* + 0.4Δ and *N_x_* = 50*a*. Here, *E_M_* = 0.05Δ and Γ_eff_ = 0.1Δ. We can see that the gap is not opened and the 4π period persists. (c) Energy spectrum as a function of the flux in the ring structure with μ = *−2t* + 0.8Δ and *N_x_* = 50*a*. Here, *E_M_* = −0.12Δ *>* Γ_eff_. We can see that the two states of different parity are separated in energy space. (d) An energy spectrum that is beyond the superconducting gap in the ring structure and also oscillates with the 4π period. (e) Flux-dependent DOS of the electron part (red solid line) and the hole part (blue solid line) along the odd parity state energy spectrum in panel (c). They are correlated with each other. (f) Flux-dependent DOS of the electron part (red solid line) and the hole part (blue solid line) along the energy spectrum in panel (d). They are not correlated with each other.

Although the two different structures show the same information for the interference effect, we can say that they are qualitatively different. The parity in the ring structure will not be destroyed when the parity of the whole system is conserved. However, the parity in the junction, as shown in Figure 1a, will be destroyed even if the total parity is conserved. This is attributed to the fact that there are two pairs of MFs in the system of Figure 1a, while there is only one pair of MFs in the ring structure shown in Figure 2a. If there are two pairs of MFs, the effect from the other MFs must be considered. For example, in the Josephson junction shown in Figure 1a, γ_1_ will couple with γ_2_, and γ_3_ will couple with γ_4_. The effective coupling Hamiltonian should be *H_M_* = *E_M_*_1_*i*γ_1_γ_2_ + *E_M_*_2_*i*γ_3_γ_4_, where *E_M1(2)_* represents the energy splitting between the two MFs in the left (right) superconducting wire. *E_M1(2)_* decreases exponentially with the length *L_1(2)_* of the left (right) wire: 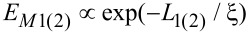 with ξ being the coherence length of the superconducting wire [42,44–45]. When effective coupling is considered in the Hamiltonian in Equation 3, the Andreev bound states would not intersect at 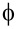 = π. In Figure 3a the red (blue) solid line shows the energy spectrum for the even (odd) parity state of the Andreev bound states formed by the MFs. Here, the wire length is infinite. Therefore, γ_1_ and γ_4_ will not destroy the parity of the Andreev bound states. When the wire length is finite (e.g., *L*_1_ = *L*_2_ = 100*a*), we can see from the dashed line that a band gap δ*E_M_* exists at 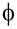 = π. Thus, the parity is destroyed, and the Josephson current has a 2π period. There is no 4π fractional Josephson Effect in the junction shown in Figure 1a.

While there are only two MFs, the parity of states will not be destroyed even if we consider the effective coupling induced by the finite length of the wire. In this case, the total low-energy effective Hamiltonian can be described as follows:

[5]
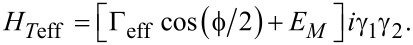


Here, Γ_eff_ is the effective coupling between the two MFs at the junction and *E_M_* is the energy splitting between the two MFs due to the finite length of the ring shown in Figure 2a. We can see that *E_M_* only shifts the energy of the even (odd) parity state but does not destroy the parity. In Figure 3b, we show the energy spectrum for a varying flux with μ = −2*t* + 0.4Δ and *t_c_* = 0.4*t*. Here, *E_M_* = 0.05Δ and Γ_eff_ = 0.1Δ. The two energy spectra cross over without destroying the parity of the Andreev bound states. When we consider the case of *E_M_* *>* Γ_eff_, the two states are separated. The energy spectra of the Andreev bound states shown in Figure 3c are separated and show the 4π oscillation for the ground state. In this case, we can ignore the parity conservation problem. Here, *E_M_* = −0.12Δ when the parameters are *L* = 50*a*, *t_c_* = 0.4*t* and μ = −2*t* + 0.8Δ. The analysis above indicates there are qualitative differences between one pair of MFs and two pairs of MFs. If there are two pairs of MFs, the parity of the Andreev bound states formed by the two MFs can be affected by coupling with the other MFs. However, if there is only one pair of MFs, coupling only affects the effective coupling between the two MFs but it does not destroy the parity of states. In fact, coupling induced by the finite-length effect can cause the same interference effect as in the Top-JJ of the ring structure. Both of them originate from the interference effect between the MFs.

We have shown that the 4π Josephson Effect can appear in the mesoscopic ring structure without the need to consider the parity-conserving problem. However, in this case, an unexpected coherent single electron tunneling process would occur in the mesoscopic ring structure, which is similar to the persistent current in the mesoscopic ring. It will occur in the conduction band, which lies above the superconducting gap. Figure 3d shows the energy spectrum that lies above the superconducting gap. It also oscillates with a 4π period. It is difficult to derive these two cases from the period. Here, we show that the DOS can distinguish the two different cases. The DOS caused by the MFs is parity related and has a 4π period, whereas the DOS caused by the coherent tunneling does not exhibit a parity-related oscillation. Figure 3e shows the DOS of the electron part (red solid line) and the hole part (blue solid line) of the odd parity state, respectively. We can see that they show the parity related interference pattern, where one is constructive and the other is destructive. Although the total DOS is not conserved due to the splitting of the MFs, it is qualitatively different from the DOS of the energy spectrum above the superconducting gap (Figure 3f). The DOS in Figure 3f is not parity related and shows very different oscillation behavior between the DOS of the electron part and the hole part. Thus, they can be well distinguished by considering the DOS.

### Detecting the 4π oscillation through two STM leads

**Figure 4:**
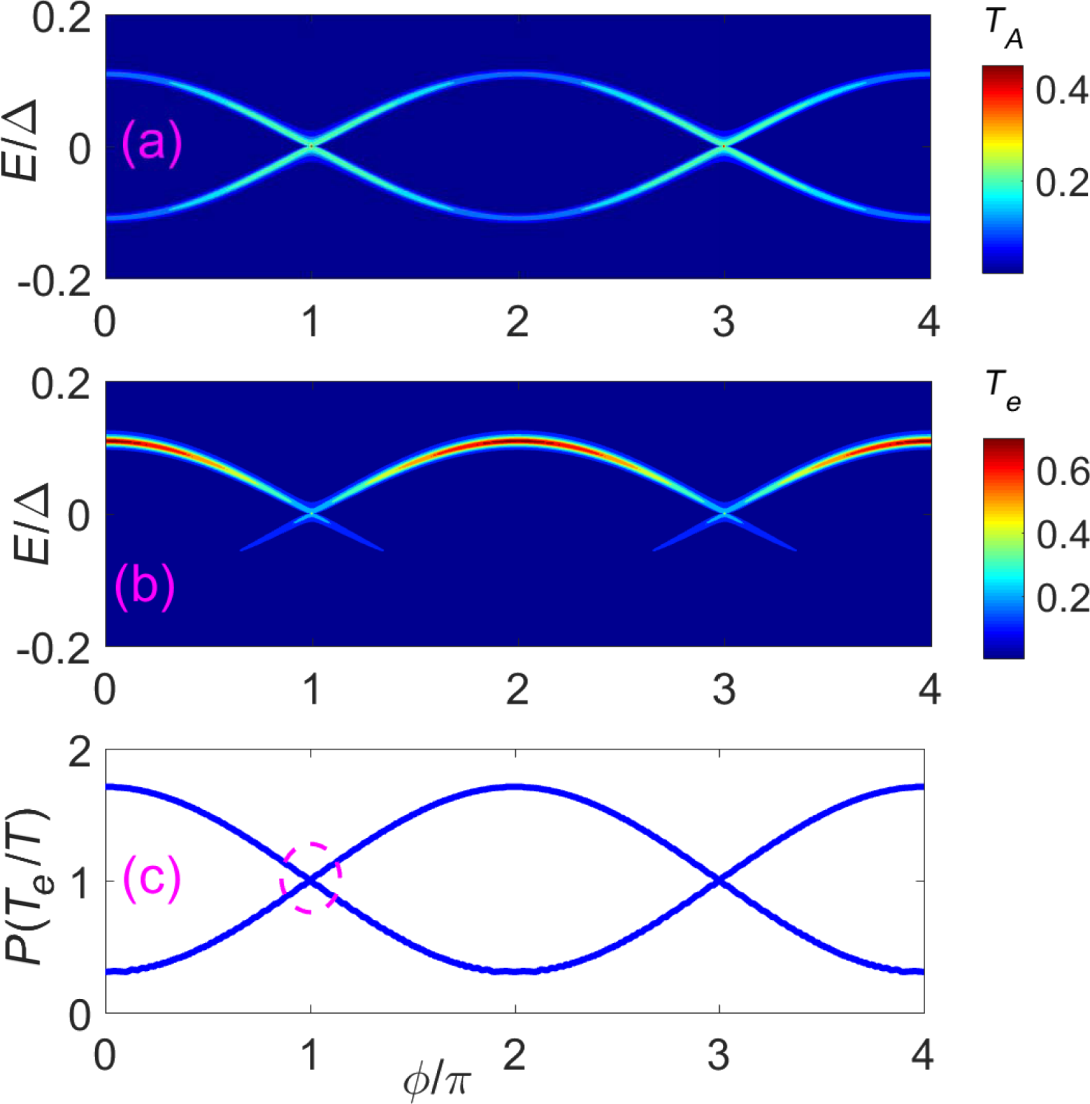
Two STM leads (or weak coupled normal leads) localized at the junction can read the putative 4π period through the differential conductance. (a) Contour plot of the Andreev reflection coefficient *T_A_* of a STM lead as a function of the flux 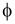 and the incident energy *E*. (b) Contour plot of the electron tunneling coefficient *T_e_* from the STM lead 1 to the STM lead 2 as function of the flux 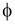 and the incident energy *E*. (c) The ratio between the peak value of *T_e_* and the peak value of *T*, here *T* = (*T_e_* + *T_A_*)/2. They show similar information of the DOS (see Figure 1c). The DOS of one energy spectrum exhibits a 4π period. However, when both spectra are considered, the period returns to 2π. In this situation, we can distinguish by the even–odd cross point as indicated by dashed circle. The parameters are *N_x_* = 200*a*, μ = −2*t*, and *V_x_* = 2Δ.

**Figure 5:**
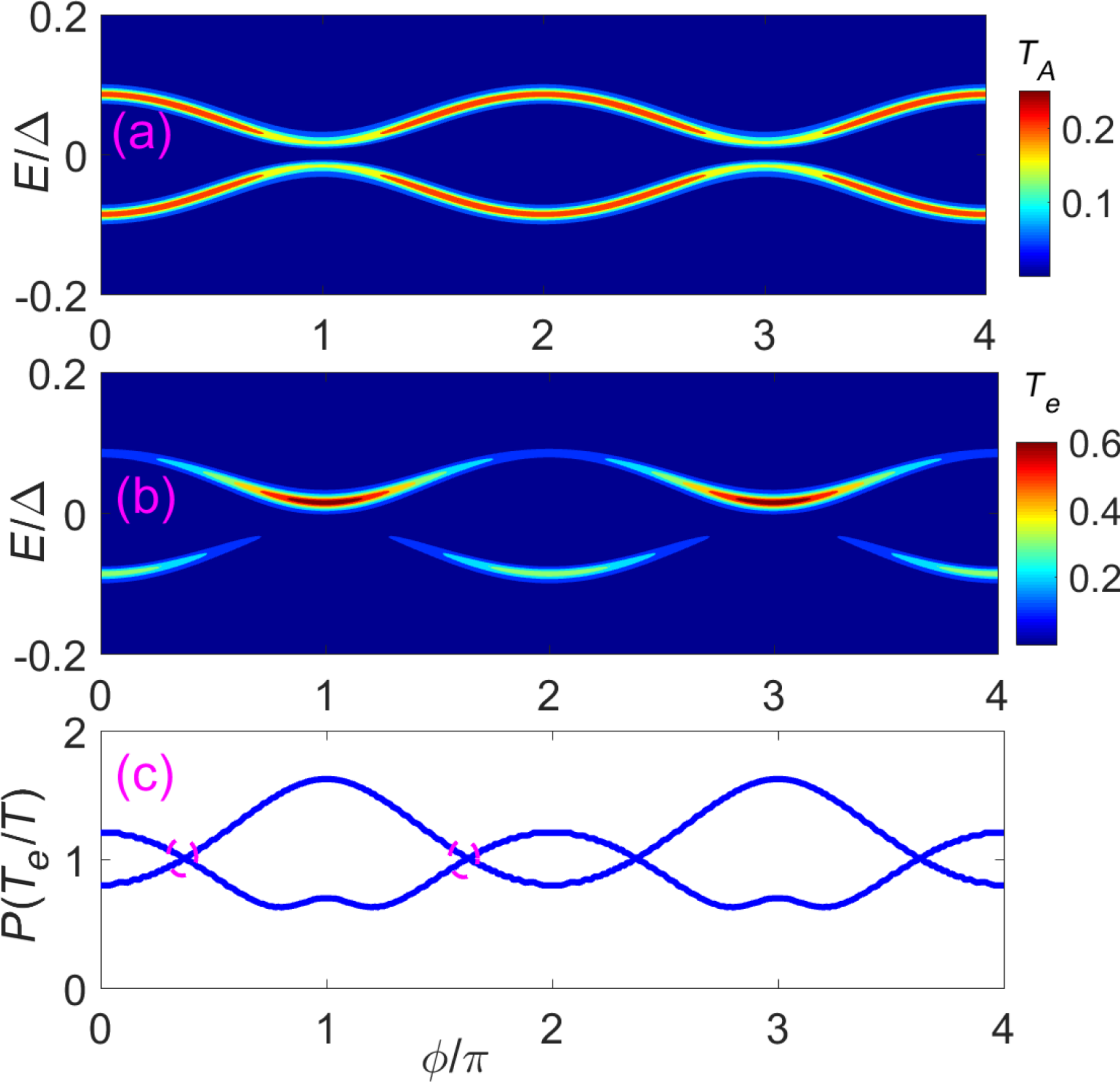
The case for two accidentally touching Andreev bound states. (a) Contour plot of the Andreev Reflection coefficient *T_A_* as a function of flux 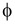 and the incident energy *E*. (b) Contour plot of the electron tunneling coefficient *T_e_* from the STM lead 1 to the STM lead 2 as a funciton of the flux 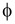 and the incident energy *E*. For both cases, the period is 2π. (c) The ratio between the peak value of *T_e_* and the peak value of *T*, here *T* = (*T_e_* + *T_A_*)/2. They yield similar information as the DOS for the trivial states. The obvious characteristic is that they will intersect an even number of times or not at all in a 2π period as indicated by dashed circles. The parameters are *N_x_* = 200*a*, μ = −2*t* + 5.7Δ, and *V_x_* = 2Δ.

In the last section, we have shown that the main features of the DOS for the nontrivial Andreev bound states are parity-correlated with a 4π period, which is very different from the trivial case. Next, we describe how the parity-correlated 4π period of the DOS can be detected. The intuitive approach would be to put a STM lead (normal lead) to detect the local DOS. However, this does not work. In our previous paper [46], we studied the conductance at the junction with a single STM lead. A butterfly-pattern conductance caused by nontrivial Andreev bound states would be observed as we vary the flux, which is distinct from the conductance of a single impurity state localized at the junction. Hence, the butterfly pattern can be regarded as a unique property of the nontrivial Andreev bound states. Figure 4a shows the same butterfly-pattern conductance. However, the peak value of the butterfly for each parity-conserved energy spectrum has a 2π period instead of a 4π period. The reason for this is that a single STM lead can only read the information of the local DOS via Andreev reflection. Although the numerical results in Figure 4 and Figure 5 are calculated using recursive Greens function methods, the relation between Andreev reflection and DOS can be obtained using a simplified effective model. These two methods are consistent with each other. The calculation of the Andreev reflection coefficient through the effective model can be found in the appendix or in [47], and can be expressed simply as


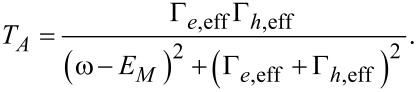


Here, Γ*_e_*_,eff_ is the effective self-energy of the electron part of the leads, Γ*_h_*_,eff_ is the effective self-energy of the hole part of the leads, and *E_M_* is the coupling energy of the two MFs. 
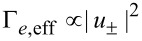
 = 
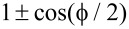
 is proportional to the DOS of the electron part, and 
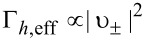
 = 
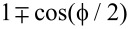
 is proportional to the DOS of the hole part. Thus, the Andreev reflection reveals the combined DOS of the electron and the hole parts, which is a 2π period. It cannot reveal the DOS of the electron (hole) part separately. In addition, we can see that if the two MFs are decoupled from each other, |*u*_±_|^2^ = |υ_±_|^2^ and *T_A_* shows the well-known resonant Andreev reflection caused by the MFs.

To detect the local DOS of the electron part or the hole part, we need additional information beyond the Andreev reflection process. Thus, it is necessary to add another STM lead to detect the electron transmission or the crossed Andreev reflection between the two leads [47–48]. This can directly reveal the information of the DOS. During this process, the electron tunneling coefficient between the two leads is


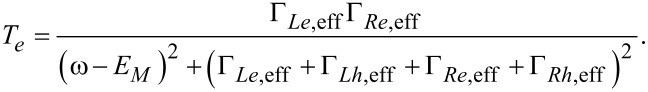


Here, 
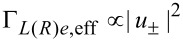
 = 
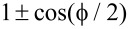
 is the effective electron part self-energy of the STM lead L(R), which is proportional to the local DOS for the electron part. In Figure 4b, we show the contour plot of *T_e_* as a function of the flux 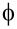 and the incident energy *E*. We can see that the peak value of the tunneling coefficient *T_e_* is proportional to 
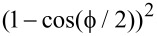
, i.e., the square of the DOS of the electron part. In addition, there is a sharp peak located at 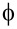 = (2*n* + 1)π. The peak appears due to the overlap between the two energy spectra at the position 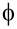 = (2*n* + 1)π. This is a main feature of nontrivial Andreev bound states: The two energy spectra intersect with each other. A better way to distinguish the information of DOS is to combine both the Andreev reflection and the electron transmission. In Figure 4c, we plot the ratio between the peak value of *T_e_* and the peak value of *T*. Here *T* = (*T_e_* + *T_A_*)/2 is the average tunneling coefficient of the Andreev reflection and electron transmission. We can see that this ratio is very similar to the DOS. One spectrum is proportional to 
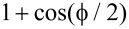
, while the other one is proportional to 
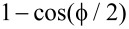
. Thus, combining the electron transmission and the Andreev reflection process can reveal the parity-correlated 4π oscillation of the DOS.

The tunneling coefficients show a very different behavior when we use two normal leads to detect the trivial Andreev bound states. Figure 5a shows the Andreev reflection coefficient as a function of the flux 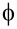, while Figure 5b displays the evolution of the electron transmission coefficient with varying 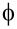. The obvious 2π period can be easily distinguished using the tunneling coefficient of electron transmission. However, the trivial Andreev bound states are susceptible to the external circumstance. When the two leads are attached to the junction, the two accidently touched trivial states will not overlap. In addition, the DOS will also be affected by the lead contact. The DOS will show a small variance when the coupling strength of the leads changes. As shown in Figure 5c, the ratio *T_e_*/*T* changes a little compared to the DOS of trivial Andreev bound states. However, two properties are preserved: First, the period is still 2π and can be described as *a* + *b*cos(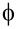); second, both electron DOS and hole DOS are generally unrelated, which strongly indicates that the two Andreev bound states are not clearly correlated with each other. Thus, the nontrivial Andreev bound states can be distinguished from the trivial Andreev bound states by combining both the electron transmission process and the Andreev reflection process.

Finally, we want to point out that the actual period in Figure 4c returns to 2π when both parity states are considered. However, we can still distinguish the trivial Andreev bound states and the nontrivial Andreev bound states by the DOS. As shown in Figure 4c, the DOS of nontrivial Andreev bound states is 
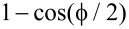
 for an even parity state and 
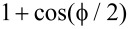
 for an odd parity state. The plots of the DOS for different parity states would overlap once (see the dashed circle in Figure 4c). While the DOS of the trivial Andreev bound states is *a* + *b*cos(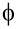), the plot of the DOS for trivial states would overlap with zero or even times in a 2π period as indicated by dashed circle in Figure 5(c). This is decided by the functional properties of cos(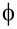) and cos(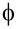/2). This kind of even–odd crossing would not be affected by a small variance of the DOS. Thus, in general, we can still distinguish the trivial states and nontrivial states through the even–odd crossing of the DOS in a 2π period.

## Conclusion

We have studied the interference effect of two MFs in a topological Josephson junction and a ring structure system. We show that the 4π Josephson effect originates from the interference between the two MFs, and so does the DOS of the nontrivial Andreev bound states. Thus, detecting the behavior of the DOS can directly reveal the nature of the fractional Josephson effect. The trivial states, which behave like the nontrivial Andreev bound states, are considered in the paper. Although it is difficult to distinguish the two cases through the supercurrent and the energy spectrum, it can be well separated through the DOS. We suggest that the DOS can be detected using two normal leads, i.e., STM leads. With the two leads, we can obtain the electron transmission process beyond the Andreev tunneling process. Then, the information of the DOS can be derived by combining the two processes.

## Appendix

### Effective Hamiltonian and effective current formula

In the main text we calculate the tunneling coefficients using the recursive Green function method. To better understanding the numerical results, we obtain the analytical results using the effective Hamiltonian and scattering matrices. The effective Hamiltonian *H*_eff_ = *H_N_* + *H_M_* + *H_T_* can be formulated as follows:

[6]
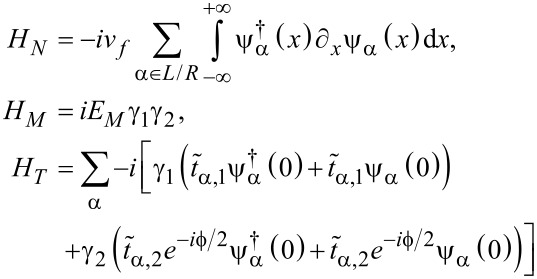


Here, *H_N_* is the Hamiltonian of the left and right normal leads; ψ*_L(R)_* denotes a fermion operator of the left (right) normal lead, and *v_f_* is the corresponding Fermi velocity of the leads. *H_M_* describes the two coupled MFs, where *E_M_* is the coupling strength between the two MF end states γ_1_ and γ_2_. The coupling between the leads and the MFs is described by *H_T_*, where the coupling strengths are represented by 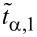 and 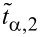, respectively.

To calculate the scattering matrix of the system, we perform a transformation first. Considering that a single MF is just half of an ordinary fermion state, we can change the MF representation into the fermion representation γ_1_ = *d* + *d*^†^, γ_2_ = *i*(*d* − *d*^†^). Then, *H_M_* and *H_T_* are changed to:

[7]
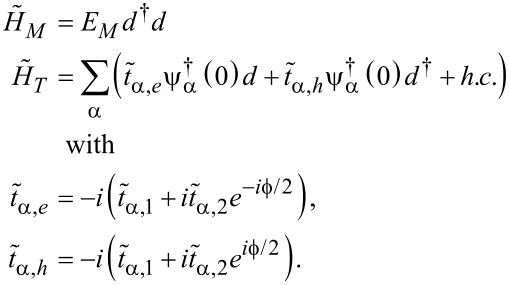


Next, we can formulate the scattering matrix in a model-independent form,

[8]
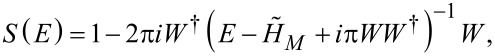


with *W* the matrix that describes the coupling between the scattering region and the leads:


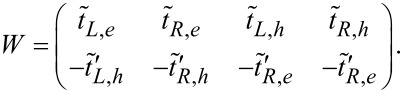


In general, we can write the approximation as:


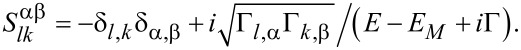


Here, Γ*_l,α_* is the self-energy of the α part of the lead *l*, which is renormalized by the local DOS of the two coupled MFs. Furthermore, it is proportional to the local DOS of the α part of the two coupled MFs. Thus, using the scattering matrix we can find the information of the local DOS. However, only a single tunneling process cannot provide all information. We need more tunneling processes, and the two leads are necessary here. There are three tunneling processes in such a two-lead setup: the Andreev reflection, the crossed Andreev reflection, and the electron transmission. We consider a symmetric connection case and simplify the result. For this condition, the coefficient of the Andreev reflection is the same as the coefficient of the crossed Andreev reflection. Then, the current for lead 1 is *I*_1_ = (2*T_A_* × *V*_1_ + (*T_A_* + *T_e_*)(*V*_1_ − *V*_2_))*e*/*h* and the current for lead 2 is *I*_2_ = (−2*T_A_* × *V*_2_ + (*T_e_* − *T_A_*)(*V*_1_ − *V*_2_))*e*/*h*. Thus, *T_e_* and *T_A_* can be obtained using the current relation.
